# Peer Review of “Performance Drift in Machine Learning Models for Cardiac Surgery Risk Prediction: Retrospective Analysis”

**DOI:** 10.2196/60428

**Published:** 2024-06-12

**Authors:** Anonymous

**Keywords:** cardiac surgery, artificial intelligence, risk prediction, machine learning, operative mortality, data set drift, performance drift, national data set, adult, data, cardiac, surgery, cardiology, heart, risk, prediction, United Kingdom, mortality, performance, model

*This is the peer-review report for “Performance Drift in Machine Learning Models for Cardiac Surgery Risk Prediction: Retrospective Analysis.”*

## Round 1 Review

### General Comments

Overall, I think this is a really interesting paper [1]. It is a concept I had never heard of, and I can see very clearly how this is an important consideration. I also think the authors have done excellently to consider a host of different aspects, including feature importance change, beyond the most obvious measurements.

### Specific Comments

#### Abstract

1. “It has been suggested that using Machine Learning (ML) techniques, a branch of Artificial intelligence (AI), may improve the accuracy of risk prediction.” Improve them over what? Specify what the status quo is with regard to first principles and data-driven modeling. This statement is also repeated in the first line of the introduction—what is “conventional” about these models?

2. “five ML mortality prediction models”—it should be highlighted that these are novel models that you have developed for this paper.

3. “geometric average results of all metrics”—it is not all metrics, just the 5 that you have calculated. It is better to just say here “a novel metric called the CEM” or something.

#### Introduction

Why is data set drift a problem? I think you could do more here to highlight how important this is to an audience who might not be dealing with the data themselves and, thus, might not naturally think of examples: for example, changes in treatment guidelines, demographics, new risk factors emerging, or changes in coding practices. You could mention “new” comorbidities such as long COVID.

#### Methods

1. Could the same individuals be in both the training and validation set and holdout set, if they had multiple surgeries? If so, this may have introduced some bias into the performance estimates. I do not think you need to redo the analyses, but if you can highlight the degree of overlap, then that would be good. Otherwise, say it was not possible and list it as a limitation.

2. “As a sensitivity analysis, we excluded the True Negative Rate from the performance evaluation, by calculating the F1 score.” This sentence does not quite make sense to me. The *F*_1_-score is based on the sensitivity (true negative rate) and the precision (positive predictive value), right? It does not exclude the true negative rate per se; it just does not use it.
